# Disordered Minds, Disordered Meals: When Emotions Masquerade as Hunger in Eating Disorders—A Systematic Review

**DOI:** 10.3390/nu18091350

**Published:** 2026-04-24

**Authors:** Giuseppe Marano, Marco Lanzetta, Camilla Scialpi, Antonio Sottile, Oksana Di Giacomi, Caterina Brisi, Gianandrea Traversi, Osvaldo Mazza, Esmeralda Capristo, Gabriele Sani, Eleonora Gaetani, Marianna Mazza

**Affiliations:** 1 Department of Neuroscience, Head-Neck and Chest, Section of Psychiatry, Fondazione Policlinico Universitario Agostino Gemelli IRCCS, Largo Agostino Gemelli 8, 00168 Rome, Italy; 2 Department of Neuroscience, Section of Psychiatry, Università Cattolica del Sacro Cuore, 00168 Rome, Italy; 3 Unit of Medical Genetics, Department of Laboratory Medicine, Ospedale Isola Tiberina-Gemelli Isola, 00186 Rome, Italy; 4 Spine Surgery Department, Bambino Gesù Children’s Hospital IRCCS, 00168 Rome, Italy; osvaldo.mazza1973@hotmail.it; 5 Department of Translational Medicine and Surgery, Fondazione Policlinico Universitario Agostino Gemelli IRCCS, 00168 Rome, Italyeleonora.gaetani@unicatt.it (E.G.); 6 Unit of Internal Medicine, Cristo Re Hospital, 00167 Rome, Italy

**Keywords:** eating disorders, emotion dysregulation, interoceptive awareness, bodily signal processing, hunger–satiety cues, transdiagnostic framework

## Abstract

Emotion dysregulation and altered interoceptive processing are increasingly recognized as core processes in eating disorder (ED) psychopathology. Difficulties in identifying, tolerating, and regulating negative emotional states may interact with reduced trust in bodily signals and distorted interpretation of hunger and satiety cues, contributing to maladaptive eating behaviors. The aim of this PRISMA-guided qualitative systematic review was to synthesize original empirical evidence on the interaction between emotion dysregulation and interoceptive processing in EDs, with particular attention to how emotional distress may be misinterpreted as physical hunger or food-related urges. A systematic search of PubMed and Scopus identified 78 records; after removal of duplicates and screening procedures, 24 original empirical studies were included in the qualitative synthesis. Across ED presentations, emotion dysregulation consistently emerged as a central transdiagnostic process associated with symptom severity, impulsivity, maladaptive eating behaviors, and related risk outcomes. Interoceptive alterations were widely reported, particularly involving reduced body trust and distorted interpretation of internal sensations rather than a uniform deficit in interoceptive sensitivity. The reviewed studies also showed that emotional states and cognitive expectations may shape the appraisal of hunger and satiety cues, contributing to the misinterpretation of emotional distress as physiological need across restrictive, binge-purge, and binge-eating presentations. The findings support a close interplay between emotion dysregulation and altered interoceptive processing in EDs and highlight the clinical relevance of interventions that integrate emotion regulation and interoceptive awareness to promote more adaptive eating behaviors.

## 1. Introduction

Eating disorders (EDs) are complex psychiatric conditions characterized by persistent disturbances in eating behavior and body-related experience, associated with significant psychological distress and functional impairment. Beyond weight- and shape-related concerns, a growing body of empirical evidence highlights the central role of emotional and bodily processes in ED psychopathology. This perspective is in line with embodied and psychosomatic models of psychopathology, which conceptualize eating disorder symptoms as expressions of dysregulated emotional processing mediated through bodily experience [1,2].

Difficulties in identifying, tolerating, and regulating negative emotional states, such as anxiety, sadness, or shame, have been consistently linked to disordered eating behaviors across diagnostic categories [3,4]. From a developmental perspective, disturbances in emotional processing and bodily experience have also been shown to emerge early in life, contributing to identity-related vulnerabilities and psychosomatic expressions of ED psychopathology across childhood and adolescence [5].

Emotion dysregulation has increasingly been conceptualized as a transdiagnostic mechanism underlying diverse ED presentations, including anorexia nervosa (AN), bulimia nervosa (BN), binge-eating disorder (BED), and subclinical disordered eating. Consistent with transdiagnostic models of psychopathology, deficits in emotion regulation have been proposed as core mechanisms underlying multiple forms of psychopathology, including EDs [6,7].

EDs have been shown to be associated with widespread neural and cognitive alterations extending beyond observable eating behaviors, particularly in brain systems involved in emotion processing, self-regulation, and interoceptive integration [8].

Empirical studies conducted in both clinical and non-clinical samples show that emotion regulation difficulties are associated with greater symptom severity, impulsivity, and maladaptive behaviors, including nonsuicidal self-injury and substance use [9,10,11]. Network-based investigations further support this transdiagnostic perspective by identifying emotion dysregulation as a highly central process linking eating pathology with broader domains of psychological distress [4,12,13]. Qualitative evidence complements these findings, indicating that ED behaviors are often described as attempts to cope with overwhelming, poorly differentiated, or unexpressed emotional states [3,14].

Alongside emotional dysregulation, increasing attention has been directed toward alterations in interoceptive awareness, defined as the perception and interpretation of internal bodily signals. Interoception plays a central role in emotional awareness and self-regulation, providing a physiological substrate through which emotions are experienced and interpreted [15,16].

From a developmental perspective, disrupted profiles of interoceptive functioning in children and adolescents have been associated with emotional difficulties and broader mental health outcomes, suggesting that alterations in interoceptive processing may emerge early and contribute to vulnerability to psychopathology [17].

Individuals with EDs frequently report reduced trust in bodily sensations and difficulties interpreting internal cues related to hunger, satiety, and affective states [18,19]. Importantly, emerging evidence suggests that interoceptive dysfunction in EDs is primarily qualitative, involving distorted interpretation and reduced bodily trust rather than a uniform reduction in sensory sensitivity [19,20,21]. Such alterations have been associated with clinically relevant outcomes, including suicidal ideation and impairments in social and occupational functioning [18,20], and more pronounced interoceptive deficits have been observed in particularly severe clinical subgroups, such as individuals with EDs and a history of suicide attempts [22]. Disturbances in bodily awareness have also been reported in individuals recovered from EDs, suggesting persistence beyond behavioral symptom remission [23,24].

**Figure 1:**
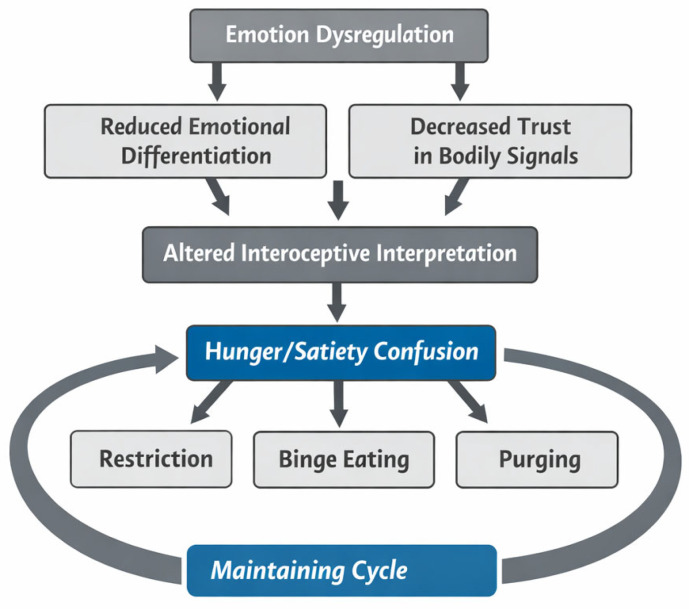
Integrated conceptual model of emotion dysregulation and altered interoceptive processing in eating disorders. Note: Emotion dysregulation and altered interoceptive processing are conceptualized as bidirectionally related processes. Difficulties in identifying and regulating emotional states may reduce trust in bodily signals and distort the interpretation of internal sensations such as hunger and satiety; conversely, altered interoceptive interpretation may impair emotional differentiation and regulation. These reciprocal alterations may promote maladaptive eating behaviors, including restriction, binge eating, and purging, within a self-maintaining cycle.

Figure 1 summarizes a conceptual framework integrating emotion dysregulation and interoceptive alterations as reciprocally interacting processes contributing to ED psychopathology. In this perspective, altered interoceptive interpretation may both result from and contribute to emotion dysregulation, reinforcing maladaptive eating behaviors over time.

Within this framework, the present PRISMA-guided qualitative systematic review aims to synthesize original empirical evidence on the interaction between emotion dysregulation and interoceptive processing in EDs, with particular attention to the misinterpretation of hunger and satiety signals and their clinical implications. 

The aim of this PRISMA-guided qualitative systematic review was to synthesize original empirical evidence on the relationship between emotion dysregulation and interoceptive processing in eating disorders. More specifically, the review sought to examine how difficulties in emotional regulation and altered bodily signal processing may interact to promote the misinterpretation of hunger and satiety cues and contribute to maladaptive eating behaviors across AN, BN, BED, and subclinical disordered eating presentations.

## 2. Materials and Methods

### 2.1. Study Design

This study was conducted as a PRISMA-guided qualitative systematic review in accordance with the Preferred Reporting Items for Systematic Reviews and Meta-Analyses (PRISMA) 2020 guidelines [25]. In line with PRISMA recommendations, only original empirical studies were considered as primary evidence. Systematic reviews, scoping reviews, theoretical papers, and commentaries were excluded from the pool of primary studies and were used solely to contextualize findings in the Introduction and Discussion.

The objective was to synthesize original empirical evidence examining the relationship between emotion dysregulation, interoceptive awareness, and ED psychopathology. 

### 2.2. Search Strategy

A literature search was conducted in the electronic databases PubMed and Scopus. Search terms covered three core domains: (1) eating disorders, (2) emotion regulation and related constructs (e.g., alexithymia), and (3) interoception and bodily awareness. Database-specific Boolean search strings were applied to the Title and Abstract fields. Searches were restricted to peer-reviewed articles written in English. No restrictions were imposed on the year of publication in order to capture both foundational and contemporary contributions. 

The full search strategies for each database are reported in Appendix A.

### 2.3. Eligibility Criteria

Studies were included if they met all the following criteria: employed an original empirical design (quantitative, observational, experimental, or intervention-based); examined individuals with a diagnosed ED or subclinical disordered eating; assessed emotion dysregulation, emotional awareness, or related constructs (e.g., alexithymia); investigated interoceptive awareness, bodily signal processing, or explicitly linked emotional processes to eating-related behaviors; were published in peer-reviewed journals.

Studies were excluded if they were systematic reviews, scoping reviews, narrative reviews, theoretical papers, commentaries, case reports, conference abstracts, or book chapters; if they were not published as peer-reviewed journal articles; if they focused exclusively on obesity without assessment of ED psychopathology; or if they did not include emotional or interoceptive constructs relevant to the review aims.

### 2.4. Study Selection

The study selection process followed the PRISMA guidelines and was conducted in multiple stages. A search of PubMed and Scopus identified a total of 78 records potentially relevant to the aims of the review. After removing 14 duplicate records, 64 unique articles remained and were screened at the title and abstract level.

**Figure 2:**
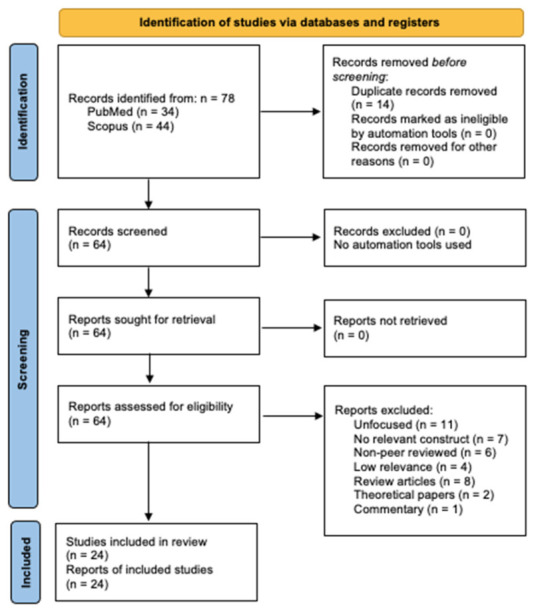
PRISMA flow diagram of the inclusion process for this review.

During this first screening phase, 39 records were excluded for the following reasons: absence of an ED or disordered eating population (n = 11); lack of relevant emotional regulation or interoceptive constructs (n = 7); publication type not consistent with the predefined eligibility criteria for peer-reviewed journal articles, including book chapters and editorial materials (n = 6); insufficient relevance to the aims of the review (n = 4), meaning that the article addressed eating-related, body-related, or affective topics but did not specifically examine the relationship between emotional dysregulation or related emotional constructs and interoceptive processing in EDs or subclinical disordered eating; systematic or scoping reviews (n = 8), theoretical papers (n = 2), and commentary articles (n = 1). Following full-text screening, 25 articles were assessed for eligibility. One study was excluded because the full text could not be retrieved through institutional or database access. An attempt was made to contact the authors to obtain a copy, but the article remained unavailable for inclusion. As a result, 24 original empirical studies were included in the qualitative synthesis. The PRISMA flow diagram illustrating the study selection process is shown in Figure 2.

### 2.5. Data Extraction and Synthesis

From each included study, data were extracted on authorship, year of publication, study design, population characteristics, ED diagnosis or disordered eating status, measures of emotion dysregulation and/or interoceptive awareness, and key findings relevant to the review aims.

In line with PRISMA recommendations, the review adopted a conservative approach, prioritizing methodological clarity and conceptual relevance over exhaustive database coverage. Studies were organized into thematic domains addressing emotion dysregulation, interoceptive awareness, and their interaction in ED psychopathology.

## 3. Results

To improve conceptual clarity, the findings are presented according to the framework summarized in Figure 1. Specifically, the narrative synthesis is organized around four interrelated domains: emotion dysregulation as a core transdiagnostic process; altered interoceptive awareness and reduced trust in bodily signals; the interaction between emotional and interoceptive processes; the misinterpretation of hunger and satiety cues as a pathway to maladaptive eating behaviors. Intervention findings are then summarized as clinically relevant attempts to target these mechanisms.

### 3.1. Characteristics of Included Studies

The qualitative synthesis included 24 original empirical studies published between 2014 and 2025. The studies employed heterogeneous methodologies, including cross-sectional observational designs, experimental paradigms, intervention-based studies, network analyses, and one randomized controlled trial [26].

Study samples comprised children, adolescents, and adults, with the majority of investigations focusing on female participants, in line with the epidemiology of ED. The included studies examined a range of ED presentations, including AN, BN, BED, mixed ED samples, and subclinical disordered eating, particularly in community and college populations [4,13].

Several studies adopted case–control designs, including healthy control groups, allowing for direct comparisons of emotional and interoceptive processes between individuals with and without EDs (e.g., Torres et al., 2024 [27]; Knejzlíková et al., 2021 [28]). An overview of study characteristics is reported in Table 1.

### 3.2. Emotion Dysregulation as a Core Behavioral Process

Across the included studies, emotion dysregulation was systematically assessed using validated self-report measures, network analyses, and qualitative approaches. Network-based studies consistently identified indices of emotion regulation difficulties as highly central variables within symptom networks, linking eating pathology with impulsivity, body dissatisfaction, and other psychological symptoms [4,12,13].

In clinical samples, higher levels of emotion dysregulation were associated with greater ED symptom severity, impulsivity, and co-occurring risk behaviors, including non-suicidal self-injury and substance-related problems [9,10].

Studies focusing on binge eating reported significant associations between emotion regulation difficulties, emotional eating motivation, and binge frequency or severity [32]. Qualitative and mixed-methods investigations described ED behaviors as being used in the context of unrecognized, overwhelming, or poorly differentiated emotional states [3].

Intervention studies reported pre-post reduction in ED symptoms, alongside improvements in emotion regulation measures following emotion-focused or body-oriented treatments [26,31].

### 3.3. Interoceptive Awareness and Bodily Signal Processing

Alterations in interoceptive awareness and bodily signal processing were reported across ED diagnoses. Several studies documented reduced interoceptive awareness, altered interpretation of bodily sensations, or lower trust in internal signals, particularly in individuals with AN [18,19,20].

Experimental and clinical studies showed that emotional states influenced the perception of physiological sensations, including bodily cues related to internal states [19,28]. Lower scores on the *Trusting* and *Self-Regulation* subscales of the Multidimensional Assessment of Interoceptive Awareness (MAIA) were associated with clinically relevant outcomes, including suicidal ideation in clinical ED samples [18] and impairments in work and social functioning in individuals with AN [20]. Disturbances in bodily awareness were also observed in individuals recovered from EDs, indicating the persistence of altered body-related processing beyond behavioral symptom remission [23].

### 3.4. Interactions Between Emotion Dysregulation and Interoception

Several studies directly examined the relationship between emotion dysregulation and interoceptive processes. Structural equation modeling demonstrated that impairments in interoceptive awareness were associated with emotional vulnerability and indirectly related to non-suicidal self-injury [10], while network analyses identified emotion regulation difficulties as central nodes linking ED symptoms and maladaptive behaviors [4,13]. Other studies reported that difficulties in perceiving bodily cues were associated with reduced emotional differentiation and greater emotion regulation difficulties, which, in turn, were linked to ED symptoms [21,36,37].

In contrast, in studies focusing on binge eating, emotion regulation variables and emotional eating motivation showed stronger associations with binge behavior than interoceptive awareness measures [32].

### 3.5. Behavioral Effects of Body-Oriented and Emotion-Focused Interventions

Intervention-based studies examined treatments explicitly targeting emotional regulation and/or bodily awareness. Body-oriented approaches, including dance movement therapy and sensory-based strategies, were associated with reductions in emotional dysregulation and alexithymia, alongside improvements in interoceptive awareness and ED symptom severity [14,31,35,38,39].

Similarly, integrative recovery protocols and mindfulness-informed interventions reported increases in body awareness and self-regulatory capacities, accompanied by reductions in ED-related symptoms [26,37,40].

### 3.6. Misinterpretation of Hunger and Satiety Signals

Within the conceptual model outlined in Figure 1, the misinterpretation of hunger and satiety signals can be understood as the behavioral expression of the interaction between emotional dysregulation and altered interoceptive processing.

Several studies examined how individuals with EDs perceive and interpret hunger and satiety signals. Findings indicated that hunger-related bodily cues were frequently associated with emotional, cognitive, or predictive processes, rather than being evaluated solely as physiological signals.

In AN, emotional states were shown to modulate the perception of physiological sensations, with hunger cues often experienced as aversive, ambiguous, or misattributed rather than as neutral bodily signals [19,37].

In BN, findings supported an altered interoceptive inference process, whereby internal signals such as hunger and fullness were interpreted according to prior expectations and predictive beliefs rather than current physiological input [30].

In binge-eating presentations, difficulties in recognizing satiety cues appeared to be primarily related to emotion regulation deficits and emotional eating motivations, with interoceptive awareness playing a less direct role in driving eating behavior [32].

### 3.7. Summary of Results

Overall, the reviewed evidence supports the conceptual model presented in Figure 1. Across diagnostic groups, emotion dysregulation emerged as a central transdiagnostic process associated with symptom severity, impulsivity, and maladaptive eating behaviors. Interoceptive disturbances were consistently described not as a simple generalized deficit in sensory detection, but rather as qualitative alterations involving reduced trust in bodily sensations, impaired interpretation of internal states, and difficulty distinguishing emotional arousal from physiological cues.

The interaction between these two domains appears clinically meaningful. Difficulties in identifying and regulating emotional states may increase reliance on bodily signals that are themselves poorly trusted or misinterpreted, thereby favoring maladaptive behaviors such as restriction, binge eating, or purging. In this sense, the available evidence suggests that emotional distress may be translated into distorted bodily experience, including the misreading of hunger and satiety cues.

Taken together, the findings indicate that ED psychopathology may be sustained by a self-reinforcing cycle in which emotion dysregulation and altered interoceptive processing mutually amplify one another. Table 2 provides a concise study-by-study summary of the main findings included in the review.

## 4. Discussion

The present review integrated original empirical evidence examining how emotion dysregulation and interoceptive awareness jointly contribute to ED psychopathology.

In line with developmental and psychosomatic perspectives, these findings support a view of EDs as conditions involving the interaction between emotional processing and bodily experience across the lifespan [5]. Although the studies included in this review did not directly compare the relative influence of these processes with cognitive distortions related to weight and shape, the available evidence suggests that ED psychopathology is more comprehensively understood within a multifactorial framework that includes cognitive, emotional, and interoceptive dimensions.

### 4.1. Emotion Dysregulation as a Core Transdiagnostic Mechanism

Across the included studies, emotion dysregulation consistently emerged as a central process associated with ED symptoms and related maladaptive behaviors. Network analyses identified difficulties in emotion regulation as highly central variables linking eating pathology with impulsivity, body dissatisfaction, and non-suicidal self-injury within diagnostic groups [4,12,13]. These patterns are consistent with transdiagnostic models of psychopathology, which conceptualize emotion dysregulation as a shared vulnerability underlying heterogeneous symptom expressions [41]. Comparable associations between emotion regulation difficulties, bodily confidence, and ED symptom severity have also been reported in AN samples, highlighting the relevance of emotional awareness for self–body integration [34].

From a clinical perspective, studies conducted in ED samples showed that higher levels of emotion dysregulation were associated with greater symptom severity, functional impairment, and comorbid risk behaviors, including substance use and non-suicidal self-injury [9,10]. Qualitative and mixed-methods evidence further indicated that ED behaviors are often described by patients as attempts to manage intense, poorly differentiated, or unexpressed emotional states [3,12].

Together, these findings suggest that routine clinical assessment in ED populations should extend beyond eating-specific symptoms to include systematic evaluation of emotion regulation capacities, particularly emotional awareness, tolerance of negative affect, and regulatory strategies. Difficulties in these domains appear closely linked to symptom maintenance and comorbid risk behaviors across ED presentations, supporting their relevance as both assessment targets and potential treatment targets.

### 4.2. Interoceptive Awareness and Bodily Experience in EDs

The reviewed studies consistently reported alterations in interoceptive awareness, particularly involving reduced trust in bodily sensations and difficulties interpreting internal signals. Alterations in the monitoring and interpretation of internal bodily signals may compromise emotional identification, contributing to alexithymic features and broader emotion regulation difficulties in EDs [42]. Importantly, the findings indicate that interoceptive dysfunction in EDs is better characterized by qualitative alterations in the interpretation and trust of bodily signals, rather than by a generalized reduction in sensory sensitivity [18,19,20]. In AN, emotional states were shown to influence the perception of physiological sensations, including bodily needs, indicating that hunger and other internal cues may be experienced as ambiguous, aversive, or threatening [19]. Reduced confidence in bodily signals was associated with clinically relevant outcomes, such as suicidal ideation and impairments in social and occupational functioning [18,20]. Consistent with these findings, elevated interoceptive deficits have also been reported in ED patients with a history of suicide attempts, further underscoring the clinical salience of bodily mistrust [22].

Notably, altered bodily awareness was also observed in individuals recovered from EDs, suggesting persistence of interoceptive disturbances beyond behavioral symptom remission [23]. Similar alterations in emotional and physiological responding have been documented in adolescent girls with restrictive anorexia nervosa, indicating that disruptions in bodily and emotional processing may emerge early during the disorder [28].

These findings are consistent with embodied and interoceptive models of psychopathology, which highlight the role of bodily signals in emotional awareness and self-regulation and align with recent mappings of body-related experimental research in anorexia nervosa [43]. Clinically, they suggest that treatment approaches focusing exclusively on cognitive restructuring may be insufficient when trust in bodily sensations and interoceptive interpretation remain compromised.

### 4.3. Interactions Between Emotion Dysregulation and Interoception

Several studies explicitly examined how emotion dysregulation and interoceptive processes interact. Structural equation modeling and network analyses showed that interoceptive impairments were associated with emotional vulnerability and maladaptive behaviors, including nonsuicidal self-injury [10]. Other studies reported that difficulties in perceiving bodily cues were linked to reduced emotional differentiation, which in turn was associated with greater emotion regulation difficulties and ED symptoms [36,37]. From a neurodevelopmental perspective, evidence suggests that early alterations in reward processing, emotion regulation, and sensitivity to internal cues may constitute precursors of binge-eating behaviors in children and adolescents, with environmental factors such as exposure to ultra-processed foods potentially amplifying these vulnerabilities [44].

However, the evidence also suggests that the relative contribution of these processes may vary by ED presentation. In binge eating, emotion regulation difficulties and emotional eating motivations showed stronger associations with binge behavior than interoceptive awareness alone [32]. This pattern supports process-based models in which emotion dysregulation may act as a more proximal driver of behavior, with interoceptive alterations shaping how emotional states are experienced and interpreted.

Clinically, these findings highlight the potential utility of integrated interventions that simultaneously address emotional regulation skills and bodily awareness, rather than treating these domains in isolation. Recent advances in digital phenotyping and machine learning further support this integrated perspective, showing that multimodal behavioral signals, including handwriting, graphomotor patterns, and vocal features, can capture alterations in emotional regulation and internal state processing, offering objective markers of psychopathological conditions and their underlying embodied mechanisms [45,46,47].

### 4.4. Misinterpretation of Hunger and Satiety Signals

A central theme emerging from the reviewed studies concerns the distorted appraisal of hunger and satiety cues. Rather than reflecting simple deficits in interoceptive sensitivity, EDs appear to involve systematic misinterpretations of bodily signals, influenced by emotional states and cognitive expectations. Consistent with this view, hunger has been conceptualized as the outcome of an interpretative interoceptive process rather than a direct readout of physiological need, such that bodily signals of energy deficit may be experienced as ambiguous, emotionally charged, or misattributed to affective distress in EDs [48].

In AN, hunger-related sensations were modulated by affective states and frequently experienced as aversive or threatening [19,37]. In BN, findings supported a predictive or inferential account of interoception, whereby internal signals such as hunger and fullness are interpreted according to prior expectations rather than current physiological input [30]. In binge-eating presentations, hunger sensations were closely linked to emotion regulation difficulties and emotional eating motivations, suggesting that internal cues may be experienced as emotional distress rather than physiological need [32].

From a clinical perspective, these findings suggest that improving patients’ ability to differentiate emotional states from physiological hunger and to re-establish trust in bodily signals may be critical treatment targets.

### 4.5. Implications for Treatment

Intervention studies included in this review provide preliminary but converging support for the clinical relevance of emotion-focused and body-oriented treatments in EDs. Across different therapeutic formats, interventions explicitly targeting emotional regulation capacities and bodily awareness were associated with improvements in emotion regulation, reductions in alexithymia, enhanced interoceptive awareness, and decreases in ED symptom severity [14,26,31,35]. Although the number of such studies remains limited, these findings are consistent with theoretical models emphasizing embodied emotional processes in ED psychopathology, which highlight emotion regulation, interoceptive awareness, and related self-regulatory capacities as key mechanisms of change [40].

From a process-based perspective, treatments that enhance emotional awareness, tolerance of negative affect, and regulatory flexibility may directly address core mechanisms sustaining ED psychopathology. Emotion-focused approaches aim to improve patients’ ability to identify, label, and tolerate internal emotional states, thereby reducing the need to externalize distress through eating-related behaviors. In this context, difficulties in emotion differentiation and regulation, which emerged as highly central processes in network analyses, represent clinically meaningful treatment targets rather than epiphenomena of ED symptoms.

Body-oriented and interoception-focused interventions appear particularly relevant in addressing the qualitative alterations in bodily experience observed in EDs. Rather than restoring interoceptive sensitivity per se, these approaches emphasize rebuilding trust in bodily signals, improving the interpretation of internal sensations, and fostering a more coherent sense of embodied self-awareness, consistent with predictive and inferential models of interoception [49,50].

Interventions incorporating mindfulness, movement-based and dance-oriented therapies, sensory modulation, and interoceptive exposure have been associated with improvements in body awareness and trust, emotion regulation capacities, and broader self-regulatory functioning, alongside reductions in ED symptomatology [26,31,33,38,39]. Such findings align with embodied models of psychopathology, which posit that emotional regulation and bodily awareness are deeply interconnected processes, as described in neurobiological models of interoception and affective regulation [51].

Importantly, the reviewed evidence suggests that treatment implications may differ across ED presentations. In AN, where hunger and other bodily cues are often experienced as aversive or threatening, interventions targeting bodily mistrust and emotional avoidance may be particularly indicated. In BN, approaches informed by interoceptive inference models may help patients recalibrate predictive beliefs about internal states, reducing reliance on maladaptive eating behaviors driven by distorted bodily expectations. In binge-eating presentations, emotion-focused interventions addressing emotional eating motivations and distress tolerance appear especially relevant, given the established role of emotional eating as a maladaptive regulation strategy [52], as emotion regulation difficulties may represent a more proximal driver of binge behavior than interoceptive deficits alone.

These findings support the integration of emotion-focused and body-oriented components within established evidence-based treatments, rather than their consideration as alternative or adjunctive approaches. For example, incorporating interoceptive awareness training, emotion differentiation exercises, and body-based self-regulation strategies into cognitive–behavioral or integrative treatment frameworks may enhance treatment efficacy by addressing underlying embodied processes that sustain ED behaviors. Preliminary evidence from randomized and open-label interventions suggests that such integrative approaches may improve both psychological well-being and functional outcomes, although the evidence base remains limited.

Future intervention research should prioritize dismantling and mediation designs to clarify whether improvements in emotion regulation and interoceptive awareness represent active mechanisms of change. Identifying which patients benefit most from emotion-focused versus body-oriented strategies may further inform personalized treatment planning. These approaches are consistent with models emphasizing the bidirectional relationship between interoceptive processing and emotional experience [16].

Overall, the findings of this review underscore the clinical importance of moving beyond symptom-focused interventions toward mechanism-informed treatments that explicitly target the interaction between emotional regulation and bodily experience in eating disorders.

**Figure 3:**
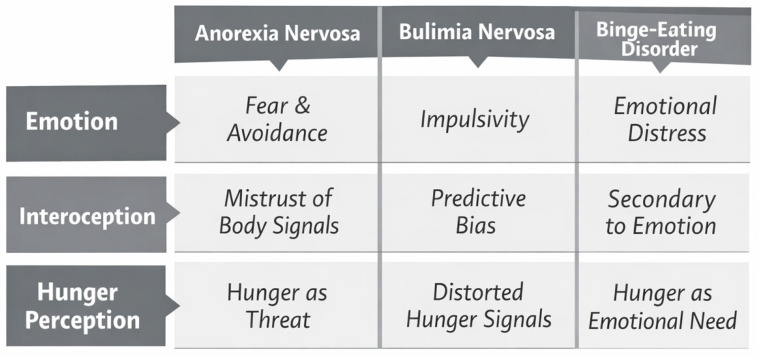
Differential patterns of interaction between emotion dysregulation and interoceptive processing across eating disorder presentations. Note. In AN, bodily signals such as hunger are often experienced as aversive or threatening in the context of emotional avoidance and reduced bodily trust. In BN, altered interoceptive inference biases the interpretation of internal cues toward predictive beliefs rather than physiological states. In BED, emotional distress and regulation difficulties appear to play a more proximal role, with hunger sensations frequently reflecting emotional rather than physiological needs.

Given the heterogeneity of ED presentations, Figure 3 illustrates differential patterns of interaction between emotion regulation and interoceptive processing across AN, BN, and BED. These differential patterns suggest that emotion-focused and body-oriented interventions may need to be tailored according to the predominant emotional–interoceptive profile characterizing each ED presentation.

### 4.6. Limitations and Future Research Perspectives

Several limitations of the present review should be acknowledged. First, although the review followed PRISMA guidelines and focused exclusively on original empirical studies, the majority of included investigations adopted cross-sectional designs. This methodological predominance limits the possibility of drawing causal inferences regarding the directionality of the relationships between emotion dysregulation, interoceptive alterations, and ED behaviors. While the converging evidence supports a close association between these processes, longitudinal studies are needed to clarify whether impairments in emotion regulation and interoceptive awareness represent vulnerability factors, maintaining mechanisms, or consequences of ED psychopathology.

Second, most studies relied primarily on self-report measures to assess emotion regulation and interoceptive awareness. Although widely validated instruments such as the Difficulties in Emotion Regulation Scale and the Multidimensional Assessment of Interoceptive Awareness were frequently employed, self-report approaches may be influenced by limited insight, alexithymic traits, and response biases, which are particularly relevant in ED populations. The reliance on subjective measures may therefore underestimate the complexity of interoceptive processing, which involves both perceptual and inferential components. Future research would benefit from integrating multimethod approaches, including behavioral tasks, experimental paradigms, and physiological indices of interoception, to complement self-reported experiences.

Third, substantial heterogeneity was observed across studies in terms of diagnostic composition, age range, clinical severity, and assessment tools. Many investigations included mixed ED samples or subclinical disordered eating populations, potentially limiting the generalizability of findings to specific diagnostic subtypes. Moreover, female participants were markedly overrepresented, reflecting the epidemiology of EDs but constraining conclusions regarding male and gender-diverse populations. These factors highlight the need for more diagnostically stratified and demographically diverse samples in future studies.

Fourth, although this review aimed to examine the interaction between emotion dysregulation and interoceptive processing, the included studies varied considerably in how these constructs were operationalized. In several cases, interoception was assessed indirectly through body trust or bodily awareness subscales rather than through comprehensive models of interoceptive inference. This conceptual variability may partly account for inconsistencies across findings and underscores the importance of adopting clearer theoretical frameworks when investigating embodied emotional processes in EDs.

Despite these limitations, the present review provides a methodologically coherent synthesis of original empirical evidence highlighting the intertwined roles of emotion dysregulation and altered interoceptive processing in ED psychopathology. Future research should prioritize longitudinal and process-based designs to determine whether changes in emotion regulation and interoceptive awareness mediate symptom trajectories and treatment outcomes. In addition, intervention studies explicitly targeting both emotional regulation capacities and bodily awareness are needed to clarify their causal relevance and therapeutic potential. Such approaches may help refine personalized, mechanism-informed treatments that address the embodied emotional processes sustaining ED behaviors.

## 5. Conclusions

This review synthesized evidence from 24 original empirical studies examining the relationship between emotion dysregulation, interoceptive awareness, and ED psychopathology. By focusing exclusively on original empirical research, the review provides a methodologically coherent overview of behavioral and embodied processes implicated across ED presentations.

Across the reviewed studies, emotion dysregulation consistently emerged as a central, transdiagnostic process, associated with ED symptoms and maladaptive behaviors across AN, BN, BED, and subclinical disordered eating [4,12,13]. Difficulties in identifying, tolerating, and regulating negative emotional states were linked to greater symptom severity, impulsivity, and related risk behaviors [9,10].

In parallel, the evidence highlighted widespread alterations in interoceptive processing, particularly involving reduced trust in bodily signals and distorted interpretation of internal sensations, rather than uniform deficits in interoceptive sensitivity [18,19,20]. Importantly, similar disturbances were also observed in individuals recovered from EDs, suggesting persistence of altered body-related processing beyond behavioral symptom remission [23].

A converging finding across diagnoses concerned the misinterpretation of hunger and satiety cues, whereby emotional states and cognitive expectations shape the appraisal of bodily signals. In AN, emotional modulation influenced the perception of physiological sensations, rendering hunger cues aversive or ambiguous [19,37]. In BN, altered interoceptive inference processes biased the interpretation of internal cues toward prior expectations rather than physiological states [30]. In binge-eating presentations, hunger sensations were frequently linked to emotion regulation difficulties and emotional eating motivations rather than to physiological need [32].

Clinically, these findings underscore the importance of integrating emotion regulation and interoceptive targets within assessment and treatment, rather than focusing exclusively on behavioral normalization. Evidence from intervention studies suggests that emotion-focused and body-oriented approaches, including mindfulness-informed and movement-based interventions, may improve both emotional regulation and bodily awareness, with associated reductions in ED symptomatology [26,31,35].

At the same time, the conclusions of this review should be interpreted in light of limitations in the existing literature, including the predominance of cross-sectional designs, reliance on self-report measures, and diagnostic and methodological heterogeneity. Future research should prioritize longitudinal, multimethod, and process-based designs to clarify causal pathways and determine whether changes in emotion regulation and interoceptive awareness mediate treatment outcomes [10,36].

In conclusion, the present review highlights that EDs cannot be fully understood or effectively treated without addressing the interconnected roles of emotion dysregulation, interoceptive awareness, and the interpretation of bodily signals, positioning embodied emotional processes as central targets for advancing both research and clinical practice.

## Figures and Tables

**Table 1:** Characteristics of the studies included in this review.

Author(s), Year	Study Design	Sample	ED Diagnosis/Population	Measures of Emotion Dysregulation/Interoception
Cook-Cottone et al., 2025 [26]	Randomized controlled trial	n = 277; 100% female	ED (mixed diagnoses)	Emotion regulation; body awareness; mindfulness
Lahaye et al., 2025 [29]	Cross-sectional	n = 20; 100% female	Adolescents with AN	Interoceptive accuracy/awareness; alexithymia
Dobyns, 2025 [14]	Pilot mixed-methods intervention	n = 27; 96% females, 4% males	BED	Emotion regulation; interoceptive awareness
Alboukrek et al., 2025 [13]	Cross-sectional network analysis	n = 943; 470 females, 473 males	Disordered eating behaviors	Emotion regulation; interoception; body dissatisfaction
Lucherini Angeletti et al., 2024 [10]	Cross-sectional (SEM)	n = 130; 100% females	AN; BN	Interoceptive awareness (EDI-2); emotion regulation
Chester et al., 2024 [30]	Cross-sectional, theory-driven	n = 61; 100% females	BN	Interoceptive inference model–based measures
Rodrigues et al., 2024 [4]	Cross-sectional network analysis	n = 159; gender NR	Disordered eating (college sample)	Emotion regulation; transdiagnostic processes
Torres et al., 2024 [27]	Case–control	n = 159; 100% female	AN	Emotion regulation; body responsiveness; well-being
Bastoni et al., 2024 [31]	Open-label intervention	n = 49; 100% female	EDs	DERS; MAIA
Iida et al., 2023 [20]	Cross-sectional	n = 36; 100% female	AN	Interoceptive awareness (EDI-2); impulse regulation
Lyvers et al., 2022 [32]	Cross-sectional	n = 532; 379 females, 152 males	BED	Alexithymia; emotion regulation; interoception
Monteleone et al., 2022 [33]	Cross-sectional network analysis	n = 303; adults; gender NR	BN; BED	Emotion regulation; childhood maltreatment
Wollast et al., 2022 [19]	Cross-sectional	n = 50; 100% females	AN	Interoceptive awareness; emotional processing
Knejzlíková et al., 2021 [28]	Experimental case–control	n = 60; adolescent girls (100% female)	Restrictive AN	Emotional responses; physiological reactivity
Perry et al., 2021 [18]	Cross-sectional	n = 102; adolescents and adults; 93% females	EDs	MAIA; body trust; suicidal ideation
Schlegl et al., 2021 [12]	Cross-sectional network analysis	n = 2535; adolescents and adults; gender NR	AN, BN	Emotion regulation; symptom networks
Monteleone et al., 2021 [34]	Cross-sectional, clinical comparative	n = 115; adults; 100% females	AN	Body confidence; emotional awareness
Bou Khalil et al., 2021 [35]	Pilot clinical intervention	n = NR; adults; gender NR	AN	Interoceptive awareness; self-disgust
Lozano-Madrid et al., 2020 [9]	Cross-sectional	n = 145; 74.5% female	ED with/without SUD	Emotion dysregulation; impulsivity; executive functions
Foye et al., 2019 [3]	Qualitative	n = 32; adults; gender NR	EDs	Emotional intelligence; emotion awareness
Smith et al., 2018 [22]	Cross-sectional	n = 192; 86% females	ED with suicide attempts	Interoceptive awareness deficits
Bernatova & Svetlak, 2017 [36]	Case–control	n = 280; young women (100% female)	EDs	Emotional and interoceptive awareness
Lattimore et al., 2017 [37]	Cross-sectional	n = 295; 100% female	ED/at-risk ED	Interoceptive awareness; mindfulness
Eshkevari et al., 2014 [23]	Cross-sectional	n = 167; adults; 100% females	EDs/Recovered EDs	Body image disturbance; body representation

Abbreviations. AN = anorexia nervosa; BN = bulimia nervosa; BED = binge-eating disorder; ED = eating disorder; SUD = substance use disorder; DERS = Difficulties in Emotion Regulation Scale; MAIA = Multidimensional Assessment of Interoceptive Awareness; SEM = structural equation modeling; NR = not reported; HC = healthy controls.

**Table 2:** Key findings of the studies included in this review.

Author(s), Year	Key Findings
Cook-Cottone et al., 2025 [26]	The Eat–Breathe–Thrive protocol led to significant improvements in emotion regulation, body awareness, and psychological well-being in adults recovering from eating disorders.
Lahaye et al., 2025 [29]	Adolescents with anorexia nervosa showed distinct interoceptive profiles; greater interoceptive accuracy was paradoxically associated with externally oriented thinking, while interoceptive sensibility was linked to anxiety and depressive symptoms.
Dobyns, 2025 [14]	A brief body-based intervention targeting sensory modulation was associated with reductions in binge urges and perceived improvements in interoceptive awareness and emotion regulation in individuals with binge-eating disorder.
Alboukrek et al., 2025 [13]	Network analysis revealed emotion dysregulation and interoception as central nodes linking disordered eating behaviors and nonsuicidal self-injury in university students.
Lucherini Angeletti et al., 2024 [10]	Impairments in interoceptive awareness statistically mediated the relationship between early relational trauma and nonsuicidal self-injury in individuals with anorexia nervosa and bulimia nervosa.
Chester et al., 2024 [30]	Findings supported an interoceptive inference model of bulimia nervosa, suggesting altered prediction and interpretation of bodily signals related to eating behavior.
Rodrigues et al., 2024 [4]	Emotion dysregulation emerged as a highly central transdiagnostic process associated with eating psychopathology in a non-clinical college sample.
Torres et al., 2024 [27]	Lower positive body image was associated with greater emotion regulation difficulties and poorer psychological well-being in individuals with anorexia nervosa.
Bastoni et al., 2024 [31]	Dance movement therapy was associated with reductions in emotion dysregulation and alexithymia and with improvements in interoceptive awareness in patients with eating disorders.
Iida et al., 2023 [20]	Social and occupational impairments in adult women with anorexia nervosa were significantly associated with impulse regulation difficulties and reduced interoceptive awareness.
Lyvers et al., 2022 [32]	The association between alexithymia and binge eating was mediated by deficient emotion regulation rather than impaired interoceptive awareness.
Monteleone et al., 2022 [33]	Childhood maltreatment was linked to eating disorder psychopathology through networks characterized by emotion dysregulation and impulsivity in bulimia nervosa and binge-eating disorder.
Wollast et al., 2022 [19]	Individuals with anorexia nervosa showed altered subjective perception of physiological sensations modulated by emotional states, supporting an interaction between emotion processing and interoception.
Knejzlíková et al., 2021 [28]	Adolescents with restrictive anorexia nervosa exhibited altered emotional and physiological responses to mirror exposure, indicating disrupted emotional competence and bodily processing.
Perry et al., 2021 [18]	Lower interoceptive trust emerged as a significant predictor of suicidal ideation in patients with eating disorders, independent of eating pathology severity.
Schlegl et al., 2021 [12]	Symptom network analyses demonstrated that emotion regulation difficulties occupied central positions across eating disorder diagnoses and age groups.
Monteleone et al., 2021 [34]	Reduced confidence in one’s own body and self was associated with emotion-related impairments in individuals with anorexia nervosa.
Bou Khalil et al., 2021 [35]	A clinical intervention focused on interoception was associated with improvements in bodily awareness and reductions in self-disgust among patients with anorexia nervosa.
Lozano-Madrid et al., 2020 [9]	Eating disorder patients with comorbid substance use disorder showed greater impulsivity, emotional dysregulation, and altered interoceptive awareness.
Foye et al., 2019 [3]	Qualitative findings highlighted eating disorder symptoms as strategies to manage unexpressed emotions and deficits in emotional intelligence.
Smith et al., 2018 [22]	Suicide attempters with eating disorders showed significantly greater interoceptive deficits compared to non-attempters.
Bernatova & Svetlak, 2017 [36]	Emotional awareness deficits were associated with restrictive eating behaviors and reduced interoceptive awareness.
Lattimore et al., 2017 [37]	Lower acceptance of internal bodily signals and reduced interoceptive awareness were associated with greater eating disorder symptom severity.
Eshkevari et al., 2014 [23]	Persistent body image disturbance and altered bodily representation were observed after recovery from eating disorders, suggesting enduring interoceptive and body-related vulnerabilities.

## Data Availability

No new data were created.

## Appendix A

To ensure transparency and reproducibility, the complete search strategies used for each database are reported below. Searches were conducted in PubMed and Scopus, focusing on eating disorders, emotion regulation, and interoceptive processes. All searches were limited to articles published in peer-reviewed journals and written in English.

### Appendix A.1. PubMed

The following search string was applied to the Title/Abstract fields: (“eating disorder*”[Title/Abstract] OR “anorexia nervosa”[Title/Abstract] OR “bulimia nervosa”[Title/Abstract] OR “binge-eating disorder”[Title/Abstract]) AND (“emotion regulation”[Title/Abstract] OR “emotional regulation”[Title/Abstract] OR “emotion dysregulation”[Title/Abstract] OR “emotional dysregulation”[Title/Abstract]) AND

(“interoception”[Title/Abstract] OR “interoceptive awareness”[Title/Abstract] OR “interoceptive sensibility”[Title/Abstract] OR “body awareness”[Title/Abstract]

OR “bodily awareness”[Title/Abstract])

### Appendix A.2. Scopus

The following search string was applied to the Title/Abstract fields: (“eating disorder*” OR “anorexia nervosa” OR “bulimia nervosa” OR “binge-eating disorder”) AND (“emotion regulation” OR “emotional regulation” OR “emotion dysregulation” OR “emotional dysregulation”) AND (“interoception” OR “interoceptive awareness”.
